# Pericarditis with cardiac tamponade – A misleading complication of appendicitis: A rare case report

**DOI:** 10.1016/j.ijscr.2020.09.130

**Published:** 2020-09-23

**Authors:** Agnieszka Wójcik, Paweł Skorek, Dorota Palczewska, Piotr Weryński

**Affiliations:** Jagiellonian University in Cracow, Pediatric Cardiology Department, Poland

**Keywords:** Pericardial effusion, Heart tamponade, Pericarditis, Pericardectomy, Appendicitis, Case report

## Abstract

**Introduction:**

As severe complication of appendicitis as effusive pericarditis is a rare phenomenon. Taking into consideration open access data in a 50-year period we perceived three pediatric cases with pericarditis as a complication of appendicitis.

**Presentation of case:**

We report a 15-year-old boy who presented cardiac tamponade, bilateral pleural effusions, liver enlargement and ascites as a presentation of atypical appendicitis.

**Discussion:**

Even nowadays appendicitis could be difficult to diagnose in children. A delayed diagnosis could be responsible for severe morbidity and mortality.

**Conclusion:**

This report is aimed to be a reminder of serious life-threatening complications after a common abdomen condition – appendicitis.

## Highlights

• Effusive pericarditis is an extremely rare complication of appendicitis.
• It is still unknown whether pericardiocentesis or surgical approach is the treatment of choice of effusive pericarditis.
• Constrictive pericarditis normally occurs months after the initial pericarditis but occasionally it may progress rapidly.

## 1 Introduction

Appendicitis is an acute inflammatory process involving the appendix. This surgical emergency is one of the most common causes of abdominal pain, particularly between the ages of 10 and 19 years. The estimated lifetime risk is 12% for males and 25% for females [1]. However, the presentation of appendicitis could be misleading resulting in delayed diagnosis. We present a child with severe complications due to appendicitis. To provide accurate and transparent information we used SCARE Surgical Case Report Guidelines [2].

## 2 Case report

**Fig. 1:**
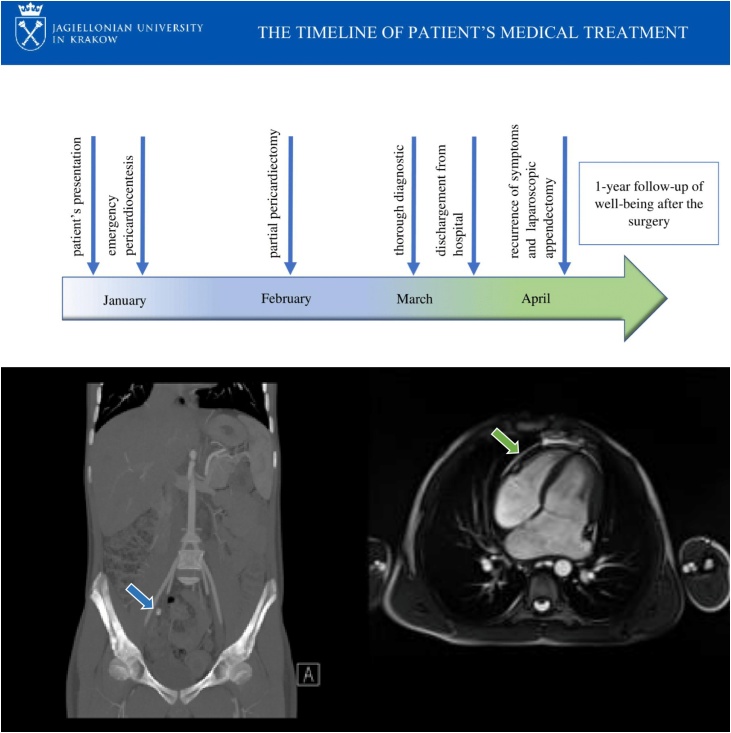
On the top the time-line of patient’s history. On the left: the contrast-enhanced computed tomogram revealed bilateral pleural effusions, fluid in peritoneal cavity and prominent appendix with surrounding inflammatory changes containing an appendicolith of size 19 mm x 12 mm (the blue arrow). On the right: heart magnetic resonance imaging with intravenous contrast medium showing a process in the pericardium (the green arrow).

A 15-year-old male presented to the Pediatric Cardiology Department in January 2019 with dyspnea at rest accompanied by chest pain. Medical interview revealed that the patient was suffering from mild abdominal pain, headache and asthenia in last few days. Medical and family history was unremarkable, he was diagnosed with allergic rhinitis and scoliosis. He has never been chronically treated. Physical examination revealed a young Caucasian who was in an obvious acute distress. Vital signs at presentation were blood pressure 110/82 mmHg, heart rate 100/min, respiratory rate 24/min, temperature 36.6 °C, oxygen saturation 99% without oxygen support, height 179 cm, weight 52 kg. Pertinent objective findings were muffled heart sounds, distention of the jugular veins, ascites with hepatomegaly and right lower quadrant tenderness on palpation with no guarding or rebound tenderness. Laboratory results showed white blood cells (WBC) 4,870/uL, hemoglobin 14 g/dL, hematocrit 44.7%, platelets 152.000/uL, glucose 124 mg/dl, international normalized ratio 1.3; C-reactive protein <5 mg/l, Troponin T 8.8 ng/l. Electrocardiogram revealed sinus tachycardia, small amplitude of QRS complex, ST elevation and depression of PQ segment in many canals. Echocardiography was performed, 40 mm pericardial fluid was depicted. Patient was diagnosed with heart tamponade. An emergency pericardiocentesis was performed, what is more, drainage tubes were inserted into the pericardial cavity. The samples of blood and purulent fluid were collected for microbiological tests. No germs developed in the bacterial examination of the pericardial fluid. Broad-spectered antibiotics were applied with colchicine, non-steroidal anti-inflammatory drugs and corticosteroids. Contrast-enhanced computed tomogram revealed pericardiac and bilateral pleural effusions, moreover an appendix with appendicolith (Fig. 1). After evaluation by a general surgeon, the appendectomy was postponed until the etiology of pericarditis would be clarified. Although, the patient was consulted by many specialists, none of them was able to disclose the ground of the patient’s disease. Many laboratory tests were conducted. Not only contagious diseases were excluded, but also lymphoma, cystic fibrosis, connective tissue disorders, immune deficiency, hypothyroidism, coeliac disease. The patient’s plasma level of antibodies was negative for numerous pathogens (aerobic and anaerobic bacteria, *Mycoplasma tuberculosis, Toxoplasma gondii*, Ebstein-Barr virus, *Cytomegalovirus*, hepatic viruses*, Chlamydia trachomatis, yersinia enterocolica,* Candida etc). Moreover, heart magnetic resonance imaging was performed and, based on the results, the patient was diagnosed with restrictive pericarditis (Fig. 1). After one month of hospitalization the inflammatory parameters increased (WBC 22.210/ul, C-reactive protein 14.1 mg/l, procalcitonin 0.18 ng/mL) additionally the hemodynamic parameters worsened. The patient subsequently underwent partial pericardectomy through anterolateral left thoracotomy performed by the an experienced professor of pediatric cardiac surgery. The clinical postoperative evolution was favorable, with normal echocardiographic aspect. The drainage tubes were extirpated. From the patient’s point of view that period of hospitalization was the most stressful. He demanded some psychologists’ help in aim to return to normal activity. 5 days after the patient had been discharged, he was readmitted to the Emergency Department, because of acute abdominal pain. Physical examination revealed right lower quadrant tenderness on palpation without vomiting, nausea or fever. The abdominal ultrasonography revealed prominent appendix with an appendicolith up to 25 mm consistent with probable acute appendicitis. In addition, echocardiography was performed and reported recurrence of pericardial effusion. After evaluation by a surgeon, the patient subsequently underwent laparoscopic appendectomy. The clinical postoperative evolution was favorable moreover the effusive fluid absorbed completely. The patient was discharged 4 days after the operation with signs of normal wound healing and continues to do well 1 year postsurgery. He does his regular check-ups in a medical clinic.

## 3 Discussion

In our case the genesis of pericardial effusion with heart tamponade and subsequent constrictive pericarditis was appendicitis. The reasons for misdiagnosis include the difficulty of interpreting the aspecific symptoms in children, the first manifestation of appendicitis was heart tamponade which is an extremely rare complication.

We searched the open access NCBI PubMed and Medline database for English literature from 1970 to march 2020 to determine the frequency and management of pericarditis as a complication of appendicitis. This delivered a total of 3 case reports [3,4]. Though, none of those cases depict an atypical presentation of appendicitis leading to over 2 months delay in treatment. It is worth to highlight that even nowadays it is sometimes challenging to diagnose appendicitis in children correctly.

The diagnosis and management of pericardial diseases remain challenging because of the vast spectrum of manifestations and the lack of clinical data on which to base guidelines by the American College of Cardiology and the American Heart Association. However, the European Society of Cardiology (ESC) published guidelines on pericardial disease in 2004. Although no criteria for the diagnosis of acute pericarditis have been established, prior studies [5] have suggested that at least 2 of the following 4 criteria should be present: (1) characteristic chest pain, (2) pericardial friction rub, (3) suggestive electrocardiographic (ECG) changes, and (4) new or worsening pericardial effusion [6,7].

Pericarditis frequently arises hematogenously from a distant focus of infection [8]. Despite the fact that the microbial flora could not be detected in patient’s samples of effusive fluid nor blood, broad-spectered antibiotics were applied.

The European Society of Cardiology guidelines recommend surgical drainage over percutaneous drainage in complicated purulent pericarditis. Through the left anterolateral thoracotomy achieves good exposure for removal of a portion of the pericardium to allow drainage [9]. The only effective therapy for constrictive pericarditis is pericardectomy with excision of the fibrotic epicardium as was performed in our patient. Controversy still exists regarding the approach and the extent of surgery thus it is unlikely to achieve consensus because of the low incidence of complicated appendicitis with purulent pericarditis.

## 4 Patient [1] perspective

The prolonged stay in the hospital caused patient’s mental crisis, fortunately he was provided with successful psychological therapy. He claimed that it was caused by the shortage in contact with acquaintances, pain caused by many surgical interventions without the knowledge of certain cause of his health problems and moreover uncertain future of his health state.

## 5 Conclusion

Pericarditis is a serious life-threatening complication thus every clinician should be aware of it. The importance of this report is that it is a reminder of rare complications after a common abdomen condition - appendicitis. This knowledge could shorten the time of hospitalization, patient’s distress and costs of unnecessary diagnostic tests. This case report highlights the importance of surgical approach in case of any complications of appendicitis.

## Declaration of Competing Interest

The authors report no declarations of interest.

## Funding

No sources of funding to declare.

## Ethical approval

This case is reported retrospectively from data obtained for clinical purposes. According to Polish law such papers as case reports did not need ethical approval by Ethics Committee of University.

## Consent

We gained the patient’s legal guardian consent to use the medical records in aim to prepare this case report.

## Author contribution

Material preparation, data collection and analysis were performed by Agnieszka Wójcik, Paweł Skorek and Dorota Palczewska. The first draft of the manuscript was written by Agnieszka Wójcik under the supervision of Piotr Weryński who has also reviewed and edited the manuscript. All authors commented on previous versions of the manuscript. All authors read and approved the final manuscript.

## Registration of research studies

N/A.

## Guarantor

Piotr Weryński.

## Provenance and peer review

Not commissioned, externally peer-reviewed.
